# Five-hundred years of medicine gone to waste? Negotiating intercultural health policy in Ecuador

**DOI:** 10.1186/1472-6963-14-S2-P70

**Published:** 2014-07-07

**Authors:** Ana Llamas-Montoya, Susannah Mayhew

**Affiliations:** 1 Department of Global Health and Development, London School of Hygiene & Tropical Medicine, London, UK

## Background

In Ecuador, access to maternity services remains significantly lower amongst indigenous women compared with non-indigenous women partly due to cultural barriers [1]. A hospital in Ecuador where the indigenous rights movement is particularly strong implemented the Vertical Birth (VB) policy to increase indigenous womens’ access to maternity services by adapting services to the local culture. This included conducting upright deliveries, introducing Traditional Birth Attendants (TBAs) and making physical adaptations to hospital facilities.

## Materials and methods

This qualitative study explored the delivery of maternity services for indigenous women in an Ecuadorian hospital. Data was collected through observation, in-depth interviews, a focus group discussion, and documentation review (e.g. hospital routine data). 40 interviews were conducted with Health Workers (HWs), managers, and policy-makers involved in maternal health at the field hospital. Data analysis was guided by grounded theory and drew heavily on concepts of “street-level bureaucracy” to explore implementation.

## Results

Actors’ values motivated their support or opposition to the VB policy and conflict ensued. Managers, policy-makers, indigenous actors and a minority of HWs supported the VB policy. For this group, improving HWs’ attitudes towards indigenous women, widely perceived as discriminatory, was key to promote equitable access to services. Most HWs initially resisted the VB policy because they were concerned about the clinical implications they attributed to the VB policy (e.g. postpartum haemorrhage and vaginal tears) and the tensions that stem from working alongside TBAs (e.g. conflicting advice given to patients). Nonetheless, TBAs played a crucial role in the VB implementation; they became women’s advocates and helped improve HWs’ attitudes towards indigenous women. HWs effectively modified the VB policy and developed coping strategies to deal with their concerns. Managers accepted these as a compromise to enable implementation. Implementation also succeeded because those supporting the VB policy were more powerful and because HWs concerns over clinical complications subsided.

## Conclusions

Whilst highly controversial, intercultural health policies such as the VB policy have the potential to improve maternity services for indigenous women.

The VB implementation was heavily shaped by HWs’ values and resulted from an ongoing negotiation between HWs and managers.

## References

[B1] Goicolea I Sebastian M Wulff M Women’s reproductive rights in the Amazon basin of Ecuador: Challenges for transforming policy into practice Health Hum Rights 2008 14 91 103 10.2307/20460105 20845861

